# Regarding diagnosis and management of Spigelian hernia

**DOI:** 10.4103/0972-9941.51319

**Published:** 2009

**Authors:** James G Bittner

**Affiliations:** Department of Surgery, Medical College of Georgia, School of Medicine, USA

Dear Editor,

I read with interest the article by Mittal and colleagues entitled “Diagnosis and management of spigelian hernia: A review of literature and our experience” published in the Journal of Minimal Access Surgery.[1] The authors have briefly reviewed a limited amount of evidence regarding Spigelian hernia characteristics, diagnosis, and management, and reported their experience with 10 patients. We recently published an extensive literature review and case series demonstrating the current clinical characteristics of and operative approaches for patients with Spigelian hernia, including varied methods of laparoscopic repair.

Since a greater proportion of patients are diagnosed with Spigelian hernia in a non-emergent setting, most undergo elective repair to prevent potential complications. Of 392 cases in the literature (2001–2007), where urgency of operation was defined, 355 patients (91%) underwent elective hernia repair.[2] Open surgical techniques remain most frequently reported; however, articles describe the utility and benefits of laparoscopic surgery.[2] Even as each surgical approach has advantages and disadvantages, surgeon preference and patient circumstance often dictate the chosen technique.

Elective open prosthetic mesh repair requires an ample incision, tissue dissection, and may involve pain and postoperative hospital stay. Advantages of a shorter hospital stay and less morbidity are associated with elective and emergent laparoscopic totally extraperitoneal (TEP) and transabdominal preperitoneal (TAPP) procedures. Laparoscopic repair of large primary or recurrent Spigelian hernias may require mesh or fascia lata graft repair; however, patients with small Spigelian hernias (≤ 2 cm) can benefit from a simple, laparoscopy-assisted transfacial suturing technique that avoids mesh insertion.[2]

Mesh-free laparoscopic transfascial suture repair, as we have described it in our recent case series, is feasible and safe for Spigelian hernias that are less than or equal to 2 cm in diameter. This novel, uncomplicated, and readily available approach for small Spigelian hernias combines the benefits of laparoscopic localization, reduction, and closure, without the morbidity and cost associated with foreign materials.[2]
